# Bilingual and Bicultural: Executive Function in Korean and American Children

**DOI:** 10.3390/bs16061032

**Published:** 2026-06-20

**Authors:** Jasmine R. Ernst, Seokyung Kim, Catherine Schaefer, Hyewon Park Choi, Stephanie M. Carlson

**Affiliations:** 1 Department of Psychological and Brain Sciences, University of Louisville, Louisville, KY 40208, USA; j.ernst@louisville.edu; 2 Institute of Child Development, University of Minnesota, Minneapolis, MN 55455, USA; 3 Department of Family Social Sciences, University of Minnesota, Minneapolis, MN 55455, USA; 4 Department of Child & Family Welfare, University of Ulsan, Ulsan 44610, Republic of Korea

**Keywords:** bilingual advantage, executive function, culture, language status, English, Korean

## Abstract

The bilingual advantage hypothesis proposes that bilingual children will display greater executive function (EF) skills compared to their monolingual peers. However, most research on this topic neglects to include monolingual children from both language groups for comparison, thus confounding language status and cultural context. To address this gap, we administered an extensive battery of EF tasks to 189 typically developing children ages 47–95 months (M_age_ = 71.47, SD = 11.68, 42.9 % Female) drawn from three language status groups: Korean-English Bilingual and English Monolingual (both in the northwestern United States) and Korean Monolingual (South Korea). Korean-English Bilingual children scored significantly higher on the EF composite than Korean Monolingual children, even after controlling for child age and verbal ability. Both English Monolingual and Korean-English Bilingual children waited significantly longer during a delay-of-gratification task than Korean Monolingual children when controlling for age and verbal ability. Korean-English Bilingual children outperformed English Monolingual and Korean Monolingual children on the Comprehensive Test of Nonverbal Intelligence. There were no significant differences between language status groups on the other individual EF tasks after adjusting for multiple comparisons. Taken together, we did not find consistent support for a bilingual advantage in EF skills: Country of residence also played a role, with children living in the United States outperforming children living in Korea in some cases.

## 1. Introduction

The cognitive consequences associated with learning multiple languages have been examined by developmental scientists for decades (Bialystok, 1999, 2017; Peal & Lambert, 1962). One area of cognition that has received substantial scientific and media attention is the relation between bilingualism and executive function (EF) skills. EF refers to the neurocognitive skills involved in goal-directed problem solving, including components of working memory, cognitive flexibility, and inhibitory control (Diamond, 2013; Miyake et al., 2000; Zelazo & Carlson, 2012, 2020). A prominent hypothesis in the field proposes a bilingual advantage, such that bilingual children are presumed to have superior EF skills compared to children who speak only one language (see Bialystok, 2015, for a review). Empirical evidence to support this hypothesis has been mixed, ranging from seminal studies finding support for a bilingual advantage (Bialystok, 1999; Bialystok & Martin, 2004; Carlson & Meltzoff, 2008) to two recent meta-analyses (Gunnerud et al., 2020; Lowe et al., 2021) finding no significant difference in EF skills for bilingual and monolingual children. Thus, in the present study, we aimed to address some of the criticisms of past studies that found support for a bilingual advantage—particularly the confounding of language status with cultural background, limited EF task coverage, and inconsistent control for verbal ability—by including three language status groups (i.e., English Monolingual, Korean Monolingual, and Korean-English Bilingual children) and controlling for verbal ability, to better isolate the association between language status and EF skills. 

### 1.1. Bilingualism and Executive Function 

Several theoretical accounts outline mechanisms for how bilingualism might be expected to enhance EF. Researchers have proposed that bilingual experience might reshape individuals’ cognitive function through experience-dependent plasticity. One account suggests that the bilingual advantage reflects improved attentional control, as bilinguals constantly regulate competing mappings between two languages and adapt the deployment of their attention (Bialystok, 2017; Bialystok & Craik, 2022). At a neurological level, another account explains that the bilingual environment strengthens the gating system in the striatum, reinforcing the basal ganglia’s capacity to route task-relevant information to the prefrontal cortex when competing signals should be resolved (Stocco et al., 2014). Supporting this, many empirical studies have reported a bilingual advantage in EF across various tasks, including both cool (i.e., tasks involving cognitively demanding but emotionally neutral contexts, such as card sorting) and hot EF measures (i.e., tasks involving emotionally charged contexts, such as waiting for food) (Bialystok & Viswanathan, 2009; Carlson & Meltzoff, 2008; Morales et al., 2013). This bilingual effect was significant over and above verbal ability or socioeconomic status (SES; e.g., Carlson & Meltzoff, 2008) and was observed across multiple cultural contexts (e.g., Bialystok & Viswanathan, 2009). A meta-analysis supported this evidence by demonstrating that bilingualism is positively associated with attentional control, working memory, and metalinguistic awareness (Adesope et al., 2010).

An increasing number of studies have challenged the bilingual advantage. In a critical review, Paap et al. (2015) noted that 80% of post-2011 research had failed to find a bilingual advantage, and argued that earlier positive findings might have been inflated by small sample sizes and due to inadequate matching on demographic variables. Subsequent studies have supported these concerns. Two recent meta-analytic studies found that the overall bilingual effect was marginal or zero when adjusting for publication bias (Gunnerud et al., 2020; Lowe et al., 2021), and a study that included over 4500 children from the ABCD dataset found no evidence for a bilingual advantage in inhibitory control, attention, or cognitive flexibility (Dick et al., 2019). This body of research suggests that if a bilingual advantage exists, it is likely to be small or variable.

### 1.2. Disentangling Bilingualism and Biculturalism

One possible reason for the inconsistent results concerning a bilingual EF advantage is cultural influence. Children who learn two languages from a young age are often also bicultural, which means that they are immersed in two cultural backgrounds. These cultural experiences—exposure to multiple social values and practices—might independently influence EF development. Tran et al. (2019) compared monolingual and bilingual preschoolers in the US, Argentina, and Vietnam, and found that bilingualism and biculturalism were each associated with distinct cognitive processes: bilingualism was linked to selective attention, switching, and inhibition, whereas biculturalism was linked to behavioral regulation and response inhibition. These findings suggest that bilinguals’ linguistic experience and cultural experience might have separable effects on different aspects of EF. However, other findings paint a less clear picture, with some studies favoring cultural over linguistic accounts (i.e., it is culture, not language status, that predicts EF) and others suggesting the opposite (i.e., it is language status, not culture, that predicts EF). For example, Samuel et al. (2018) compared EF performance of monolingual and bilingual young adults residing in Korea and Britain and found that cultural background predicted EF performance—Koreans outperforming Brits—even after controlling for bilingualism. In contrast, Riggs et al. (2014) found that bilingualism continued to predict EF in a sample of Latino children living in the US, even after accounting for biculturalism. This implies that the bilingual effect is not entirely removed by cultural influences. These mixed findings suggest that both cultural and linguistic factors may contribute to EF development, but the relative role of each remains unclear (Xie et al., 2022).

One challenge in disentangling cultural and linguistic contributions to EF is that culture itself is a multidimensional construct that researchers operationalize in different ways. In the present study, we did not measure culture directly (e.g., individualism–collectivism, parental child-rearing attitudes). Rather, we defined culture broadly to refer to a constellation of cultural influences (e.g., values, beliefs, and practices) associated with either country of residence (operationalized as the country where a child is currently being raised) or heritage country (operationalized as the country of origin where one or both parents were born). Thus, the term “heritage culture” means a broad set of cultural influences related to the family heritage background. At the same time, however, we acknowledge that this operationalization of culture does not isolate other potential factors, such as educational environments (e.g., academic expectations and education curricula) and environmental factors (e.g., socioeconomic status), which could also contribute to EF development, but were not the focus of the current study. 

The question of whether bilingualism or biculturalism explains group differences in EF performance remains unresolved, in part because of the way the studies have been designed. To date, much research compares bilinguals and monolinguals within a single country of residence and typically lacks a monolingual comparison group from a non-English language. This is important because, for bilingual children who are from immigrant families, there is no monolingual comparison group from their heritage country. Given that both the country of residence and heritage culture can shape children’s EF development, this design comparing only bilinguals and monolinguals in one country confounds bilingual children’s language status with their heritage culture. For instance, when Korean-English bilingual children in the US are compared only to English monolingual children in the US, it is unclear whether any difference reflects the linguistic experience (using two versus one language) or Korean heritage culture. Note that many Korean-English bilingual children in the US share both American (country of residence) and Korean (heritage country) cultural backgrounds, making it difficult to attribute any group differences to a single cultural source. 

To tease apart language status, heritage culture, and country of residence, studies need to include monolingual comparison groups from both the bilingual children’s country of residence and their heritage country. For example, when Korean-English bilingual children living in the US are compared to English monolinguals living in the US, the country of residence is held constant, but language status and heritage culture differ; whereas when Korean-English bilingual children living in the US are compared to Korean monolinguals living in Korea, the heritage culture is held constant, but country of residence differs, assuming all the bilingual children have Korean heritage. In this way, each comparison holds one dimension of culture constant—either the country of residence or heritage culture—while varying language status.

Only a few studies have used such a cross-national, multi-group design, and they reached opposing conclusions. Cho et al. (2021) compared Korean-English bilingual preschoolers in Canada with two monolingual groups: English monolinguals (Canada) and Korean monolinguals (South Korea). Bilingual preschoolers outperformed English monolinguals in Canada but performed similarly to Korean monolinguals in Korea on the color-word Stroop task, measuring inhibitory control. Because bilinguals performed similarly to Korean monolinguals rather than English monolinguals, the authors concluded that the heritage culture—not language status—explains the bilingual advantage. This East–West disparity in EF performance was consistent with earlier findings showing that monolingual East Asian children outperform monolingual North American peers (Oh & Lewis, 2008; Sabbagh et al., 2006; Xu et al., 2020). A similar pattern was reported more recently by Papastergiou et al. (2023), who extended this multi-group approach to Greek-English bilingual children in the UK with two monolingual control groups, comparing them with Greek monolinguals (Greece) and English monolinguals (UK). Applying k-means nearest neighbor matching on several variables (i.e., age, sex, nonverbal intelligence, SES, and language proficiency), they found that Greek-English bilinguals outperformed English monolinguals on the Arrow Stroop Task (measuring inhibition) and Backward Digit Span (measuring working memory) tasks but performed comparably to Greek monolinguals. Although the authors did not frame their findings as adjudicating between language versus culture accounts, this pattern parallels Cho et al. (2021)—bilinguals resembling monolinguals from their heritage country rather than those from their country of residence—and is therefore consistent with the heritage culture account.

S. Yang et al. (2011) reached the opposite conclusion while using a similar design. They compared Korean-English bilingual preschool children in the US with three monolingual groups: English monolinguals (US), Korean monolinguals (US), and Korean monolinguals (South Korea). Bilingual preschoolers surpassed both English monolinguals in the US and Korean monolinguals in Korea on the Attention Network Task. Because bilinguals outperformed all three monolingual groups from both their country of residence and their heritage country, the authors attributed the bilingual advantage to language status rather than their country of residence or heritage culture. These three studies reflect meaningful efforts to disentangle language and cultural factors through cross-national, multi-group designs. However, this design approach has not been widely replicated, and divergent results across the available studies have added to, rather than resolved, the uncertainty to the literature. Further replication using this design is therefore needed to clarify whether language status, country of residence, or heritage culture more strongly explains group differences in EF.

Another reason for the inconsistency across studies might come from the measurement approach. Many studies rely on a single or a few EF tasks measuring inhibitory control to test the bilingual advantage. Even when tasks measuring multiple EF components—such as inhibition and cognitive flexibility—are included, bilingual advantages tend to emerge on only some tasks, not others (e.g., Ross & Melinger, 2017), suggesting that the effect might be shaped by EF component, task, and/or sample characteristics. Furthermore, several earlier studies did not include or did not control for verbal ability (e.g., Cho et al., 2021; Riggs et al., 2014; Samuel et al., 2018; S. Yang et al., 2011), although some did (e.g., Carlson & Meltzoff, 2008; Tran et al., 2019). However, it has been found that proficiency in each individual language is often lower in bilingual children (Bialystok et al., 2010) and verbal ability has been shown to be positively correlated with EF skills (e.g., Fuhs & Day, 2011; Kuhn et al., 2016). Particularly, Carlson and Meltzoff (2008) found a bilingual advantage only when controlling for verbal ability, and interpreted this to suggest that even though raw scores on EF were not substantially different from English monolinguals, Spanish-English bilingual children of the same age were “doing more with less” language proficiency.

### 1.3. Current Study 

The current study aimed to further investigate the potential bilingual advantage in EF development while addressing the limitations of prior cross-national, multi-group studies discussed above (Cho et al., 2021; Papastergiou et al., 2023; S. Yang et al., 2011). We examined EF skills among children in three language status groups: English Monolinguals living in the US, Korean Monolinguals living in South Korea, and Korean-English Bilinguals living in the US. The inclusion of all three of these groups enabled us to parse out the role of bilingualism from cultural factors. First, comparing Korean-English Bilinguals to English Monolinguals (both in the US) tests whether bilingualism confers an advantage while holding the country of residence constant. Second, comparing Korean-English Bilinguals to Korean Monolinguals (in Korea) tests whether bilingualism confers an advantage while holding the heritage culture constant. 

Building on the design used by Cho et al. (2021), Papastergiou et al. (2023), and S. Yang et al. (2011) our study expands this prior work in four ways. First, we administered a more extensive battery of six EF tasks spanning multiple EF components, including not only cool EF but also hot EF, whereas the previous three studies measured cool EF only. Second, we controlled for children’s verbal ability, whereas Cho et al. (2021) and S. Yang et al. (2011) only reported verbal ability as descriptives. Papastergiou et al. (2023) used verbal ability for matching. This is an important analytical refinement given robust positive correlations between EF and language ability (e.g., Fuhs & Day, 2011; Kuhn et al., 2016), coupled with reports that bilingual children tend to score lower than monolingual children on vocabulary measures (e.g., Bialystok et al., 2010). Hence, adjusting for verbal ability helps to isolate the cognitive effects of bilingualism, per se. Third, we recruited a larger sample (*N* = 189; 53–69 per language group) than the previous three studies (13–34 per group), providing greater statistical power. Fourth, we expanded the age distribution of participants (47–95 months), whereas some previous studies focused on relatively narrow age windows (e.g., 3.5–5.5-year-olds in Cho et al., 2021; 4-year-olds in S. Yang et al., 2011) or covered a similar overall age span but started data collection at 5, rather than 4 years of age (i.e., 5–9-year-olds in Papastergiou et al., 2023). The broader age sampling enhances the generalizability of developmental findings, because it enables us to explore whether group differences and accounts they support, such as language status, country of residence, or heritage culture, are observed in a wider developmental period from preschool to early elementary school, during which EF matures rapidly (Zelazo & Carlson, 2012, 2020). 

We hypothesized that Korean-English Bilingual children would outperform both English Monolingual and Korean Monolingual children on EF tasks, supporting a language status account of the bilingual advantage. If, however, the two US-based groups (Korean-English Bilingual and English Monolingual) performed similarly but differently from the Korea-based group (Korean Monolingual), this would suggest that group differences are driven by country of residence rather than language status. Likewise, if the two groups with Korean heritage (Korean-English Bilingual and Korean Monolingual) performed similarly but differently from the English Monolingual group, this would lend support to a heritage culture account. 

## 2. Materials and Methods

### 2.1. Participants

Participants included 189 children living within one hour of a metropolitan area in the Pacific Northwest of the US (English monolingual participants and Korean-English bilingual participants) and in Ulsan and Seoul, South Korea (Korean monolingual participants). Children were between 47 and 95 months of age. An additional 4 children were piloted on the Attention Network Task but did not complete other assessments and were not included in the analytic sample. 

English monolingual participants (*n* = 53, 56.6% female, 43.4% male) ranged from 50 to 89 months of age (*M* = 70.34, *SD* = 11.65) and were recruited from a university participant pool of eligible families. Most of the children in the English Monolingual sample were White, non-Hispanic (79.20%), with an additional 11.30% Multiracial, 7.55% White/Hispanic, and 1.89% missing data. Incomes for English Monolingual families ranged from $25,000 to $40,000, to more than $100,000, with a median income range of $85,000 to $100,000 and 11.3% missing data. All English Monolingual children were born in the US along with 92.45% of caregivers. There was an additional 7.55% of children with at least one caregiver born in countries outside of the US, including Canada, Australia, India, and Iran. 

Korean-English bilingual participants (*n* = 67, 37.3% female, 62.7% male), ranging from 47 to 95 months in age (*M* = 71.31, *SD* = 12.97), were recruited through word of mouth and community outreach, including flyers and visits to Korean community centers and churches. Most of the children in the Korean-English bilingual sample were Asian (91.00%), with an additional 2.99% White, non-Hispanic; 1.49% Multiracial; 1.49% race not otherwise listed, and 2.99% missing data. Incomes for Korean-English bilingual families ranged from less than $10,000 to more than $100,000 with a median range of $70,000 to $85,000; 7.46% were missing data. Most of the Korean-English Bilingual children were born in the USA (80.59%), with an additional 8.96%, born in Korea, 2.99% born in Canada, and 7.46% missing data. Most of the children had at least one caregiver who was born in Korea (94.03%), the only exceptions were the 5.67% with missing data. 

Korean Monolingual participants (*n* = 69, 47.8% female, 52.2% male), ranging from 54 to 92 months in age (*M_Age_* = 72.49, *SD_Age_* = 10.39), were recruited through local daycare centers and schools. All children in the Korean Monolingual sample were Asian. Families reported monthly incomes between ₩1,500,000 (approximately $1458) to ₩2,500,000 (approximately $2431) and ₩5,000,000 or more (approximately $4861 or more); median income was in the range of ₩3,000,000 to ₩3,500,000 (approximately $2917 to $3403) and 37.7% were missing data. 

### 2.2. Procedures

The research was approved by the Institutional Review Board at the University of Washington. Trained researchers conducted sessions in both a laboratory setting and in quiet rooms in community centers. Sessions were videotaped using an in-house system or handheld cameras. In both settings, a researcher interacted one-on-one with the participant for approximately 1.5 h. Parents were permitted to view the session through a window or by sitting in the room behind the child if the family preferred. Families were compensated $20 for their time both in the US and Korea.

### 2.3. Measures

#### 2.3.1. Questionnaires

Caregivers were provided with a paper packet of questionnaires in their preferred language (English or Korean) to complete at the beginning of each session. The Family Information Questionnaire included demographic questions about the child participant (e.g., race/ethnicity, gender) and family characteristics (e.g., household income). The Language Background Questionnaire included 20 items regarding the language status of the child and their caregivers, languages spoken with friends and family, languages spoken in different contexts (e.g., school and home), and opportunities for reading. Both questionnaires were developed by the research team.

#### 2.3.2. Behavioral Assessments

Children received measures in a fixed order: Peabody Picture Vocabulary Test (PPVT), Attention Network Task (ANT), Comprehensive Test of Nonverbal Intelligence (C-TONI), Delay of Gratification (DoG), Dimensional Change Card Sort (DCCS), Miller Selective Attention Task (MSAT), and Visually Cued Recall (VCR). If the child rejected a task, the experimenter was allowed to move on and return to the task later; this happened rarely. All tasks were administered in English for the English monolingual participants, Korean for Korean monolingual participants, and in the child’s preferred language for bilingual participants. If a bilingual child appeared to be struggling to understand a task in one language, the experimenter was permitted to attempt instructions in the other language as necessary (Grosjean, 1998).

**Attention Network Task** (ANT; Rueda et al., 2004). The children’s version of the ANT was administered to assess inhibitory control. It was presented in the form of a computerized game (using a Dell Dimension 1850 with a 15-in color screen; programmed in E-Prime, Psychology Software Tools, Inc., 2001). The experimenter began the task by telling the child they were being asked to “feed a hungry fish” as quickly as possible using the left and right arrow keys on the keyboard. If the fish was facing left, the child was to press the left arrow key, and if the fish was facing right, the right arrow key. After the experimenter verbally checked the child’s understanding of the rules, the child began a practice block of 24 images with a single fish in the center of the screen (i.e., a no-flanker trial). Next, the experimenter explained to the child that sometimes other fish would also appear on the screen (i.e., a flanker trial). The child was instructed to respond only to the direction of the fish in the middle of a row of five fish. Congruent trials depicted all five fish in a row across the screen facing the same direction, whereas incongruent (attentional conflict) trials depicted the middle fish facing the opposite direction, requiring the child to inhibit the tendency to respond to the flankers instead of the target. Another practice round of 24 trials began after a verbal confirmation of rule understanding. Then, the child completed four blocks of 48 test items. Difference in reaction time between incongruent and congruent trials was used in the present analyses. 

**Comprehensive Test of Nonverbal Intelligence** (C-TONI; Hammill et al., 1997). The C-TONI was administered to assess nonverbal reasoning ability and has been validated for use with children who speak English as a second language. Children were asked to indicate which image belongs among an array of target items depicted on a page, either verbally or by pantomime. A correct response included a thematically correct item, in contrast to choices that were dissimilar or only physically similar (and thus perceptually salient). For example, one of the pictorial category trials included a frog and a rabbit as target items (both depicted on four legs). The choices for “most like” included a kangaroo, beaver on hind legs, ostrich, bear, and bird. The kangaroo was the correct thematic choice (hopping), but the bear was a perceptual distractor because it was the only one depicted with four legs. The task calls for pattern recognition but also inhibition of salient but interfering response options. To begin the task, the experimenter showed the child an array of items in a set on an easel-style book page, and said, “These two are alike in some way. Which one of these [sweeping a finger along the row of alternatives at the bottom of the page] is most like these two and should go in the empty box [pointing to the top]? Point to your answer.” The experimenter conducted three practice trials. If the child responded incorrectly, the experimenter explained which response was correct and repeated the trial. Next, the experimenter conducted the test trials without feedback, beginning with the pictorial scale. Test items were administered until the participant provided three incorrect responses within a set of five. Then, the geometric subscale (using shapes instead of pictures) was administered according to the same set of rules. Raw scores for each subtest were calculated by subtracting the number of errors from the ceiling item number, and then summed for a total C-TONI score.

**Delay of Gratification** (DoG; Mischel et al., 1989). The DoG assesses a child’s decision to delay a food reward in order to receive a greater reward after waiting. The experimenter first showed the child a hotel-style bell and explained that this was the “bring me back bell,” which could be used to call the experimenter back to the room at any time. A demonstration followed, where the experimenter stepped out of the room briefly and then returned immediately upon the child’s use of the bell. Next, the child selected a favorite treat from a few options. In the United States, these snacks included: Froot Loops, Goldfish crackers, or Teddy Grahams. In Korea, these snacks included: Goldfish crackers, fruit jellies, or raisins. If the caregiver preferred to include another snack or non-food treat, such as stickers, those options were provided. Two shallow bowls or plates were presented to the child, one with two treats and the other with ten. The experimenter then explained that she had additional work to do and needed to leave the room. However, if the child could wait in their seat without eating the treats, then they could have the larger pile when the experimenter returned. If they did not want to wait, they could ring the bell at any time, and the experimenter would return immediately. If they made this choice, however, they would only be allowed to have the smaller pile. The experimenter conducted a verbal rule check and ensured the child was comfortably seated directly in front of the treats. Then, the experimenter left the room, finding a location where they could still watch the child surreptitiously. She then waited for a period of 15 min or until the child rang the bell. Children were praised regardless of wait time and given both bowls of treats. Analyses were conducted on total wait time in minutes and on a binary score in which children who waited the full 15 min were coded 1 and those who did not were coded 0, similar to Mischel and colleagues (e.g., Sethi et al., 2000).

**Dimensional Change Card Sort** (DCCS; Zelazo, 2006). The DCCS requires children to switch flexibly between a series of sorting rules with cards. Participants were shown two recipe boxes with labels depicting simple images on the front: on the right, a card depicting a blue star, and on the left, a card depicting a red truck. Test cards matched the target cards on only one feature, depicting blue trucks and red stars. The experimenter first introduced to the “color game,” where cards with red stars belong in the box labeled with the red truck, and cards with blue trucks belong in the box labeled with the blue star. She conducted one demonstration trial and allowed the child to complete the next trial, providing correction as needed. Next, six test trials were administered, with a rule reminder before each trial. After six color trials, the experimenter announced that they would be switching to the “shape game,” where stars belong in the box marked with a star, and trucks belong in the box marked with a truck. Six trials of the shape game followed, with a rule reminder before each trial. If the child successfully sorted five or more of the six cards, the experimenter continued to the advanced level. Here, cards with and without black borders were presented to the child. The experimenter explained that if the child saw a black border, they were to sort according to the rules of the color game. If there was no black border, they were to sort according to the rules of the shape game. The experimenter conducted one demonstration trial of each type. After a verbal rule check, the child sorted 12 cards (six with borders and six without), with a rule reminder before each trial. DCCS total scores were calculated by summing correct trials.

**Miller Selective Attention Task** (MSAT; Woody-Ramsey & Miller, 1988). The MSAT task was used to measure selective attention and memory. Children were shown a picture mat with six Velcro flaps. Three flaps were labeled with a picture of a cage, and three were labeled with a picture of a house. The experimenter explained that under the flaps with cages, they would find animals, and under the flaps with houses, they would find pictures of things that belong in houses. The child was allowed to explore the mat, and then the experimenter verbally confirmed that the child understood what was under house and cage images before removing the map. The child was then shown a second mat with six flaps labeled with cages and allowed to lift the flaps to explore the animal images underneath. The experimenter asked the child to identify each animal and then removed the mat from the table. Next, she presented a mat with 12 flaps, six labeled with houses and six labeled with cages. The child was told they would need to remember where all of the animals were hidden. The experimenter set a 30 s timer, allowed the child to explore, and then presented a card depicting an animal from the mat, asking where that same animal was located. The child was allowed to open flaps until the animal was found. This procedure was conducted for six total trials. Memory recall scores, calculated as the total number of correctly identified animals on the first try, were used in the present analyses.

**Visually Cued Recall** (VCR; Zelazo et al., 2002). The VCR task was administered to assess selective attention and memory. The experimenter introduced the child to a puppet named Pat, who likes specific items. She then presented a booklet opened to a page with 12 items displayed in a grid. The experimenter explained that the child was to point only to things that Pat liked. On the first trial, the experimenter pointed to an item, saying, “On this page, Pat likes the tricycle. Can you point to the one that Pat likes?” She turned the page to reveal the next set of 12 different images, announcing two that Pat liked, then asking the child to point to them. This procedure continued, adding one item for each trial until the child was asked to remember 12 total items. The VCR highest level correct was used in the present analyses. 

**Peabody Picture Vocabulary Test-3rd Ed.** (PPVT-III; Dunn & Dunn, 1997). Receptive vocabulary was assessed using the PPVT-III in English or Korean for monolingual participants, and in both languages for bilingual participants. The experimenter showed the child a page from an easel-style book depicting four black-and-white line drawings. The experimenter conducted practice items, instructing the child to point to the image depicting the word stated by the experimenter. Once the child understood the task, the experimenter read a series of words from a standardized list and recorded the child’s responses. Testing proceeded until children erred on at least eight items from a block of 12. Because the Korean PPVT does not provide standard scores, we calculated our own age-standardized scores for this sample by regressing Korean PPVT raw scores on child age in months and saving the unstandardized residual scores. We then *z*-scored the unstandardized residuals to create an age-standardized Korean PPVT score for the sample. We used the same method for the English PPVT raw scores to create an age-standardized English PPVT. These age-standardized scores on the PPVT were included as a covariate in subsequent analyses. For bilingual children, their best score out of the two languages was used. This approach was informed by bilingual vocabulary tests, such as the Expressive One-Word Picture Vocabulary Test for Spanish-English bilinguals. Children state their preferred language; however, if the child hesitates or answers incorrectly the examiner can repeat the question in the other language. The best score out of the two is used because it best approximates general vocabulary ability.

## 3. Results

### 3.1. Descriptive Analyses 

Descriptive statistics for each language group are presented in Table 1. Available data was used for inferential statistics and missing data was dropped. Correlations among study variables are presented in Table 2. 

**EF.** We examined the correlations and conducted a Principal Components Analysis (PCA; unrotated) with the individual EF tasks to determine if an EF Composite variable was appropriate. The bivariate correlations among the C-TONI, DCCS, DoG, VCR, ANT, and MSAT scores ranged from *r*s = 0.01 to 0.29. The PCA eigenvalues and scree plot suggested a two-factor solution best fit the data; however, upon inspection of the loadings, there were no tasks that only loaded highly onto factor two. In fact, all loadings reached acceptable levels for factor one, ranging from 0.41 (MSAT) to 0.74 (DCCS). Therefore, an EF Composite score for each participant was computed by averaging *z*-scores of the C-TONI, DCCS, DoG, VCR ANT, and MSAT scores. 

**Covariate Screening.** We conducted one-way ANOVAs to identify age or verbal ability differences between language status groups. Results indicated there were no significant differences in age in months across language status groups, *F*(2186) = 0.516, *p* = 0.60, $\eta_{p}^{2}$ = 0.005. However, given that age was positively and significantly correlated with each individual EF measure as well as the EF Composite (*r*s = 0.19–0.52) we included it as a covariate in the main analyses. There were significant differences in verbal ability across language status groups, *F*(2186) = 116.8, *p* < 0.001, $\eta_{p}^{2}$ = 0.56. Pairwise comparison tests with Benjamini–Hochberg corrections applied to account for multiple comparisons (Benjamini & Hochberg, 1995) revealed the English Monolingual (*M* = 0.88, *SE* = 0.07) and Korean Monolingual group (*M* = 0.78, *SE* = 0.06) outperformed the Korean-English Bilingual group (*M* = −0.40, *SE* = 0.06, *p*s < 0.001). This screening process highlighted the importance of accounting for child age and verbal ability in subsequent analyses. 

### 3.2. Main Analyses

We first analyzed the overall EF composite, followed by separate analyses for the individual EF measures.

**Figure 1:**
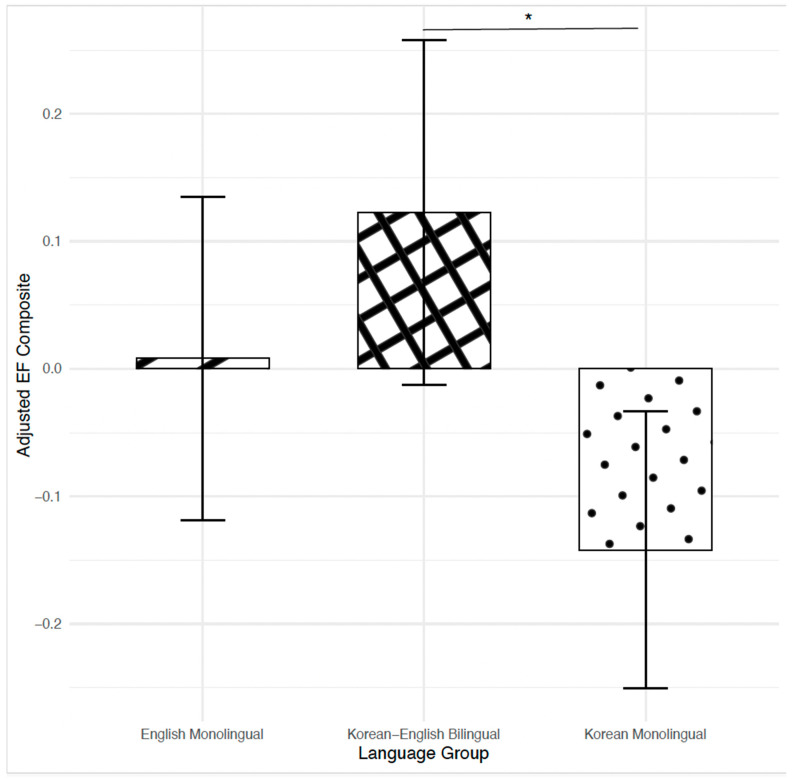
Adjusted Mean EF Composite Scores in Each Language Status Group. *Note.* Means are adjusted to control for age and verbal ability. Error bars represent 95% confidence intervals. * *p* < 0.05.

**EF Composite.** There was a significant difference between language status groups in a one-way ANCOVA predicting the EF Composite score, while controlling for child age and verbal ability, *F*(2184) = 12.44, *p* < 0.001, $\eta_{p}^{2}$ = 0.12. Pairwise comparison tests with Benjamini–Hochberg corrections revealed Korean-English Bilingual children (*M* = 0.12, *SE* = 0.07) performed better on average than the Korean Monolingual children (*M* = −0.14, *SE* = 0.06, *t* = 2.68, *p* = 0.02; see Figure 1). There was no difference between the English Monolingual and Korean-English Bilingual groups. The difference between the English Monolingual and Korean Monolingual groups was on the cusp of significance (*p* = 0.05) prior to accounting for multiple comparisons; however, the *p*-value increased after accounting for Benjamini–Hochberg corrections (*p* = 0.08).

**DoG.** Upon examination, we found the assumptions of the ANCOVA were not tenable for the DoG task. This was likely due to ceiling effects, where many children waited the entire 15 min during the DoG task. Therefore, we examined DoG as a binary outcome, such that children who waited the entire time (i.e., 15 min) received a score of 1 and everyone else received a score of 0. A logistic regression was performed to examine the effects of language group on the likelihood that participants waited the entire time in the DoG task while controlling for child age and verbal ability. Findings revealed the English Monolingual participants waited significantly longer than the Korean Monolingual participants (*B* = 1.48, *SE* = 0.41, *p* < 0.001). Additionally, the Korean-English Bilingual group waited significantly longer than the Korean Monolingual group (*B* = 1.01, *SE* = 0.52, *p* = 0.049). There was no significant difference between the English Monolingual and Korean-English Bilingual groups. 

**Figure 2:**
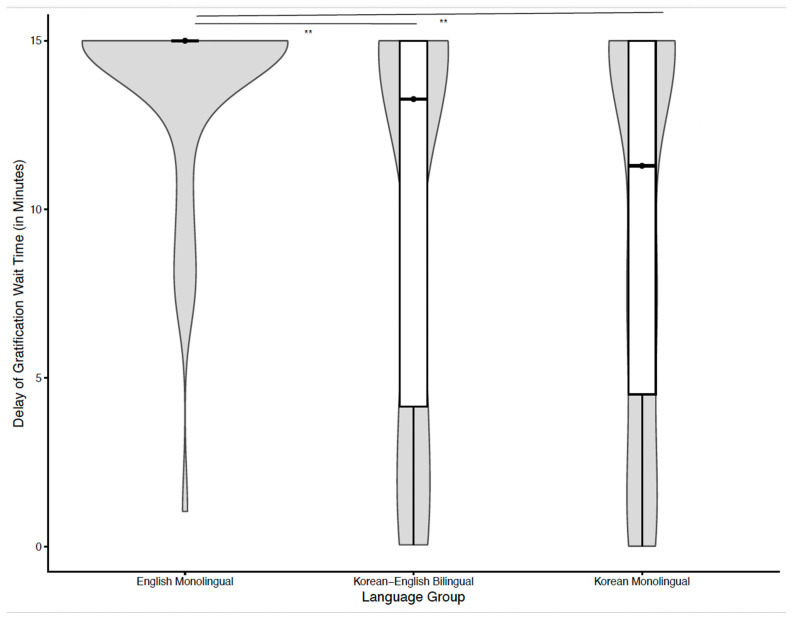
Median Delay of Gratification Wait Time in Each Language Group. *Note.* ** *p* < 0.01.

As a robustness test, we conducted a Kruskal–Wallis test as a non-parametric alternative to a one-way ANOVA; however, these analyses did not include child age or verbal ability as a covariate. We found there was a significant difference between language status groups predicting total time waited in DoG, *H*(2) = 19.54, *p* < 0.001, ε^2^ = 0.094. Pairwise comparisons tests with Benjamini–Hochberg corrections revealed that English Monolingual children (Median = 15) waited significantly longer than Korean-English Bilingual children (Median = 13.3) and Korean Monolingual children (Median = 11.3, *p*s < 0.001). There was no significant difference between Korean Monolingual and Korean-English Bilingual children (Figure 2). 

**Remaining EF Tasks.** Language status accounted for significant variance in ANT, MSAT, and DCCS scores while controlling for child age and verbal ability; however, pairwise comparisons *p*-values were greater than 0.05. There were no significant differences between language status groups in the one-way ANCOVAs predicting VCR or C-TONI scores, while controlling for child age and verbal ability (Table 3). Pairwise comparisons with Benjamini–Hochberg corrections on C-TONI scores revealed Korean-English Bilingual children (*M* = 19.7, *SE* = 0.80) performed better on average than the Korean Monolingual children (*M* = 16.8, *SE* = 0.63, *t* = 2.48, *p* = 0.02) and English Monolingual children (*M* = 16.2, *SE* = 0.74, *t* = 2.76, *p* = 0.02). There was no difference between the English Monolingual and Korean Monolingual language groups. This set of pairwise comparisons should be interpreted with caution due to the nonsignificant effect of language status in the ANCOVA. 

## 4. Discussion

The primary goal of this study was to add further evidence to the literature on bilingualism and EF and inform lively debate about the potential for a bilingual advantage. Specifically, we sought to disentangle the effect of language status from country of residence and heritage culture on childhood EF skills in Korean Monolingual children living in Korea, English Monolingual children living in the United States, and Korean-English Bilingual children living in the United States. We further aimed to address gaps in some prior literature by including a battery of EF measures and adjusting for verbal ability. We had two key findings. First, we found a partial replication of the bilingual advantage for the EF composite, such that the Korean-English Bilingual group outperformed the Korean Monolingual group, but not the English Monolingual group. Second, we found both United States-based groups (i.e., Korean-English Bilingual and English Monolingual) outperformed Korean Monolinguals based in Korea on DoG, when controlling for age and verbal ability, indicating a country of residence explanation. When age and verbal ability covariates were not included, the English Monolingual group outperformed Korean-English Bilingual and Korean Monolingual groups on DoG, suggesting culture still plays a role but through a heritage country explanation rather than country of residence. Neither set of analyses suggested DoG performance was driven primarily by language status. In isolation, it is not clear whether the EF composite findings were driven by language status, country of residence, or some combination. Taken together, the EF composite and DoG findings suggest country of residence at least partially drives the effects rather than only language status. 

### 4.1. Language Status

Korean-English Bilingual children living in the United States demonstrated better performance on EF as measured by the EF composite compared to their Korean Monolingual peers living in Korea after accounting for age and verbal ability, with a medium to large effect size. Given there was no significant difference between Korean-English Bilingual and English Monolingual children, this is not clear evidence of a bilingual advantage being driven by language status as has been previously reported (Bialystok, 1999; Bialystok & Martin, 2004; Bialystok & Senman, 2004). English Monolingual participants had non-significantly greater means than the Korean Monolingual participants on the EF Composite (*p* = 0.05 prior to Benjamini–Hochberg and *p* = 0.08 after Benjamini–Hochberg adjustment). It is likely that we did not have sufficient power to detect an effect size that was small in magnitude given that this finding was on the cusp of significance prior to adjusting *p*-values to account for multiple comparisons. Therefore, future work should consider the possibility that a significant effect could be detected with a larger sample size. 

We found some evidence of language status driving group differences for the C-TONI. Specifically, we found the Korean-English Bilingual group outperformed both monolingual groups; however, language status group was not significant in the ANCOVA. Because these differences only emerged in the planned pairwise comparisons of adjusted means it is important to interpret these findings with caution. 

Most prior work on the bilingual advantage includes two language status groups (English Monolingual and Bilingual) recruited from the same country of residence, which makes it impossible to detect the pattern of findings that emerged from our EF composite. Studies that have included all three groups have found mixed evidence on whether findings were driven by heritage culture (Cho et al., 2021) or language status (S. Yang et al., 2011); however, our findings accounted for verbal ability unlike Cho et al. (2021). Thus, our EF composite finding on its own suggests that the bilingual advantage is not entirely driven by language status because there was no significant difference between Korean-English Bilingual and English Monolingual groups. Rather, our findings indicate the EF composite findings are likely driven by country of residence or a combination of country of residence and language status. 

### 4.2. Country of Residence 

Our finding that children living in the United States waited longer in DoG than children living in Korea, after controlling for age and verbal ability, diverges from prior work. Prior studies report that children from East Asian countries outperform North American children on many EF tasks (Oh & Lewis, 2008; Sabbagh et al., 2006; Xu et al., 2020). Notably, DoG performance has been shown to be driven by cultural factors or familiarity with being asked to wait in food-based settings. Yanaoka et al. (2022) found that children in Japan delayed longer for food-based rewards compared to gifts, whereas children in the United States delayed longer for gifts than food. This finding suggests sociocultural norms can contribute to performance on lab-based delay of gratification tasks. Korean norms surrounding eating tend to prefer waiting to eat until the eldest person has taken their first bite. This is a sign of honor and respect that is rooted in Confucianism. Therefore, our finding that children in the United States groups outperformed the Korean group diverges from what one might hypothesize based on these proposed cultural mechanisms and prior empirical literature. We speculate this could be due to the DoG paradigm not invoking this cultural norm because it was framed as a choice (i.e., smaller reward now or larger reward later). This paradigm might not invoke the social cues because there is no elder that is also eating as would be the case during mealtime in the real world. This finding could also be driven partially by confounding factors we were not able to account for, such as SES. 

Beyond cultural explanations, the lower performance of Korean Monolingual children relative to US-based groups on the EF Composite and DoG task could be due, at least partially, to educational differences that our country-based proxies bundle together with culture. Early educational environments in South Korea and the United States differ in ways that could influence EF task performance. South Korea is characterized by early and intensive academic expectations, widespread participation in private education outside of school (i.e., referred to as “hagwon” in Korean), and structured early childhood curricula emphasizing early literacy and numeracy (Dittrich & Neuhaus, 2023; Choi, 2023; Lee & Park, 2025). Given this, one might predict that Korean Monolingual children would outperform US-based peers on EF tasks requiring rule-following and sustained attention. However, we observed the opposite pattern. A growing body of literature suggests that activities that respect children’s autonomy (Carlson, 2023), are based on guided play (Skene et al., 2022), scaffold mindfulness and reflection (Zelazo et al., 2018), and holistically address children’s emotional, social, and physical needs (Diamond & Ling, 2016) improve children’s EF skills and learning. By contrast, Korean Monolingual children might face elevated stress due to academic challenges and pressures from an early age (Q. Yang et al., 2026) and might have fewer opportunities for self-directed problem-solving and playful, exploratory learning to promote their EF skills. 

### 4.3. Strengths and Limitations 

The combination of theoretical and methodological rigor is a strength of the current study. Specifically, the inclusion of three language status groups from two countries enabled a more rigorous test of whether the ‘bilingual advantage’ is driven by language status or cultural factors associated with differing countries of residence and cultures inherited through their families. This expands upon prior work in the bilingual advantage literature, most of which has only included two language status groups: English-Monolingual and Bilingual. Additionally, children were administered an extensive battery of EF tasks, including both cool and hot EF assessments. 

Notably, we controlled for verbal ability, which is inconsistently included as a covariate in prior studies examining the bilingual advantage and is an area of ongoing methodological debate. We believe this covariate was important given the documented evidence of a positive association between EF and verbal skills (e.g., Fuhs & Day, 2011; Kuhn et al., 2016; Merz et al., 2019), coupled with the commonly lower vocabulary of bilingual children in either language than their monolingual peers (Bialystok et al., 2010). This differential vocabulary growth in each individual language is argued to be a characteristic of bilingualism because vocabulary is distributed across multiple languages rather than a single language. Controlling for performance on the PPVT was intended to account for the known role of language in EF development; however, we acknowledge this score might be more of a proxy for language exposure and use, rather than a true estimate of overall language ability.

Despite these strengths, there were also limitations of the current study. We cannot infer causality from this correlational study due to the non-experimental design. Additionally, we know that early experiences that shape EF skills do not occur in isolation from one another. This is important to mention because many children who are bilingual from early in life are also bicultural. Both of these experiential inputs could confer cognitive advantages by way of practice in “code switching.” Hence, it is possible that the bilingual advantage could be fully mediated by biculturalism (e.g., Spiegler & Leyendecker, 2017; Xie & Ng, 2025). It could also be that linguistic features of a language that are culturally informed are contributing to the bilingual advantage such that language is propelling a cultural effect because of the significance of language to culture.

It is also possible family factors like SES or immigration status are contributing to these findings. Prior evidence shows children from higher SES households have higher performance, on average, on EF tasks (e.g., Hackman et al., 2015; Lawson et al., 2018). Due to our inclusion of groups with differing currencies (i.e., USD and Won) it was not possible to meaningfully control for household income. Examination of family-reported income from two groups based in the United States suggests that children in the English Monolingual group were financially advantaged over the Korean-English Bilingual group. Nonetheless, the median income for both groups living in the US was above the median annual income in the US at the time of data collection ($50,303; DeNavas-Walt et al., 2009). Similarly, the median income for Korean Monolingual children living in Korea was higher than the median annual income in Korea (₩19,380,000 annually or ₩1,615,000 monthly; Korea Institute for Health and Social Affairs, 2023) at the time of data collection. This suggests many children from all three groups had a socioeconomic advantage within their country of residence. Regarding immigration status, all Korean-English bilingual children whose families reported their birthplace indicated one or both of their parents were born in Korea; most of the English Monolingual participants and their parents were born in the United States.

Further, our English Monolingual group was primarily White, non-Hispanic, which limits generalizability. Future work is needed with a more socioeconomically, racially, and ethnically diverse sample of English Monolingual children. Additionally, children in all groups were drawn from a wider age range (i.e., 4–8 years), which is why we included age as covariate in our models. It is possible that age interacts with bilingual status, such that EF advantages are stronger in younger children (when EF is emerging most rapidly) or in older bilingual children with more advanced language development. We did not have sufficient power to test this moderation in the current study, but it should be explored in future work.

There are various factors that contribute to the development of EF beyond language status or culture that we did not measure here as they were not the focus of this study (e.g., caregiving practices, Distefano et al., 2018) but could be contributing to this pattern of findings. Additionally, our conceptualization of language status groups was binary (i.e., Monolingual, Bilingual) rather than considering Monolingual and Bilingual as extremes along a continuum (Lowe et al., 2021). Future work can consider how to leverage language skill assessments to examine language status as a continuous variable. For example, it could be that bilingualism confers an advantage for EF skills only after children reach a certain knowledge or fluency threshold. 

Lastly, our design did not have the full factorial of all possible language status and country of residence groups. Ideally, future work would aim to include the following six groups: English Monolingual children living in the US, Korean Monolingual children living in the US, Korean-English Bilingual children living in the US, English Monolingual children living in Korea, Korean Monolingual children living in Korea, and Korean-English Bilingual children living in Korea. Finding large enough sample sizes in each of these groups to have sufficient power might be challenging; however, a full factorial would be the ideal non-experimental design to disentangle language status and cultural factors.

## 5. Conclusions

The current study examined whether the bilingual advantage hypothesis was supported when comparing EF performance in 4–8-year-old English Monolingual children in the United States, Korean-English Bilingual children in the United States, and Korean Monolingual Children living in South Korea. Our findings did not show consistent evidence of a bilingual advantage (e.g., Bialystok, 1999; Carlson & Meltzoff, 2008), because there was only one instance of the Korean-English Bilingual group outperforming *both* monolingual groups. We found more consistent evidence of group differences driven by country of residence (Cho et al., 2021). Interestingly, the longer delay of gratification for each United States-based group over the Korean group contradicts prior work showing children growing up in Eastern countries outperformed their Western peers on DoG tasks (Yanaoka et al., 2022). Taken together, our findings suggest that country of residence is likely playing a role in the bilingual advantage findings, but it could be working in unison with other factors like language status. Notably, there were few significant differences in most of the EF tasks administered. These findings contribute to a more nuanced, inconclusive body of evidence on whether there is truly a bilingual advantage for EF and, if so, what mechanisms are driving this advantage and for whom. 

## Figures and Tables

**Table 1:** Descriptive Statistics by Language Group.

	English Monolingual					Korean Monolingual					Korean-English Bilingual				
Variable	*n*	*M*	*SD*	Min	Max	*n*	*M*	*SD*	Min	Max	*n*	*M*	*SD*	Min	Max
Age in Months	53	70.34	11.65	50	89	69	72.49	10.39	54	92	67	71.31	12.97	47	95
Korean PPVT (raw scores)	-	-	-	-	-	69	76.07	18.66	32	104	65	31.77	16.6	9	85
English PPVT (raw scores)	53	131.45	18.79	85	161	-	-	-	-	-	66	85.8	29.8	7	144
C-TONI	52	17.21	5.47	4	30	69	17.91	5.72	4	30	65	17.74	5.31	6	30
DCCS	53	19.7	2.21	16	24	69	19.07	3.44	6	24	54	18.04	4.08	6	24
MSAT	52	2.83	1.32	1	6	69	2.7	1.53	0	6	59	2.08	1.24	0	5
VCR	53	5.55	2.08	2	10	69	5.9	2.24	0	10	60	5.22	2.28	1	10
ANT	39	−0.02	0.05	−0.18	0.12	59	−0.06	0.16	−0.95	0.05	65	−0.13	0.25	−0.99	0.12
DoG	53	13.72	2.84	1.05	15	69	9.63	5.73	0.02	15	63	9.8	5.98	0.06	15
EF Composite	53	0.18	0.44	−0.86	1.2	69	0.04	0.56	−1.34	0.98	67	−0.2	0.62	−1.95	0.8

*Note.* PPVT = Peabody Picture Vocabulary Test; C-TONI = Comprehensive Test of Nonverbal Intelligence; DCCS = Dimensional Change Card Sort; MSAT = Miller Selective Attention Task; VCR = Visually Cued Recall; ANT = Attention Network Task; DoG = Delay of Gratification.

**Table 2:** Bivariate Correlations Between Age in Months, Verbal Ability, and EF Tasks.

Variable		1	2	3	4	5	6	7	8
1.	Age in months								
2.	PPVT	0.07							
3.	C-TONI	.44 **	.17 *						
4.	DCCS	.29 **	.38 **	.29 **					
5.	MSAT	.24 **	.26 **	0.11	.22 **				
6.	VCR	.45 **	.21 **	.29 **	0.13	.21 **			
7.	ANT	.20 *	.31 **	0.09	.27 **	0.01	0.06		
8.	DoG	.19 *	.27 **	.24 **	.25 **	.17 *	0.14	.20 *	
9.	EF Composite	.52 **	.46 **	.60 **	.65 **	.52 **	.56 **	.50 **	.60 **

*Note*. PPVT = Peabody Picture Vocabulary Test; C-TONI = Comprehensive Test of Nonverbal Intelligence; DCCS = Dimensional Change Card Sort; MSAT = Miller Selective Attention Task; VCR = Visually Cued Recall; ANT = Attention Network Task; DoG = Delay of Gratification. PPVT scores are age-standardized. * *p* < 0.05. ** *p* < 0.01.

**Table 3:** Results from One-Way Analyses of Covariance and Corresponding Pairwise Comparisons on Executive Function Measures.

		ANCOVA		Pairwise Comparisons		
Measure	Parameter	*F*	*df*	Mean Difference	*SE*	*p*
ANT	Language Group	5.18 **	2, 158			
	English Monolingual vs. Korean-English Bilingual			0.01	0.05	0.826
	English Monolingual vs. Korean Monolingual			0.02	0.04	0.826
	Korean-English Bilingual vs. Korean Monolingual			0.01	0.05	0.777
DCCS	Language Group	4.28 *	2, 171			
	English Monolingual vs. Korean-English Bilingual			−0.76	0.79	0.342
	English Monolingual vs. Korean Monolingual			0.59	0.55	0.342
	Korean-English Bilingual vs. Korean Monolingual			1.35	0.73	0.203
MSAT	Language Group	5.13 **	2, 175			
	English Monolingual vs. Korean-English Bilingual			0.40	0.35	0.496
	English Monolingual vs. Korean Monolingual			0.17	0.25	0.496
	Korean-English Bilingual vs. Korean Monolingual			−0.23	0.32	0.496
C-TONI	Language Group	0.33	2, 181			
	English Monolingual vs. Korean-English Bilingual			−3.42	1.24	0.02 *
	English Monolingual vs. Korean Monolingual			−0.58	0.88	0.51
	Korean-English Bilingual vs. Korean Monolingual			2.84	1.15	0.02 *
VCR	Language Group	1.98	2, 177			
	English Monolingual vs. Korean-English Bilingual			−0.42	0.51	0.71
	English Monolingual vs. Korean Monolingual			−0.24	0.36	0.71
	Korean-English Bilingual vs. Korean Monolingual			0.179	0.47	0.71

*Note.* * *p* < 0.05. ** *p* < 0.01.

## Data Availability

Data available upon request due to restrictions (i.e., participants did not agree to public data sharing).
